# hnRNPA2/B1 Ameliorates LPS-Induced Endothelial Injury through NF-*κ*B Pathway and VE-Cadherin/*β*-Catenin Signaling Modulation In Vitro

**DOI:** 10.1155/2020/6458791

**Published:** 2020-05-30

**Authors:** Yi Chen, Dan Tang, Linjie Zhu, Tianjie Yuan, Yingfu Jiao, Hexin Yan, Weifeng Yu

**Affiliations:** Department of Anesthesiology and Critical Care Medicine, Renji Hospital, Shanghai Jiaotong University School of Medicine, Shanghai /200217, China

## Abstract

Heterogeneous nuclear ribonucleoprotein A2/B1 (hnRNPA2/B1) is a protein involved in the regulation of RNA processing, cell metabolism, migration, proliferation, and apoptosis. However, the effect of hnRNPA2/B1 on injured endothelial cells (ECs) remains unclear. We investigated the effect of hnRNPA2/B1 on lipopolysaccharide- (LPS-) induced vascular endothelial injury in human umbilical vein endothelial cells (HUVECs) and the underlying mechanisms. LPS was used to induce EC injury, and the roles of hnRNPA2/B1 in EC barrier dysfunction and inflammatory responses were measured by testing endothelial permeability and the expression of inflammatory factors after the suppression and overexpression of hnRNPA2/B1. To explore the underlying mechanism by which hnRNPA2/B1 regulates endothelial injury, we studied the VE-cadherin/*β*-catenin pathway and NF-*κ*B activation in HUVECs. The results showed that hnRNPA2/B1 was elevated in LPS-stimulated HUVECs. Moreover, knockdown of hnRNPA2/B1 aggravated endothelial injury by increasing EC permeability and promoting the secretion of the inflammatory cytokines TNF-*α*, IL-1*β*, and IL-6. Overexpression of hnRNPA2/B1 can reduce the permeability and inflammatory response of HUVEC stimulated by LPS in vitro, while increasing the expression of VE-Cadherin and *β*-catenin. Furthermore, the suppression of hnRNPA2/B1 increased the LPS-induced NF-*κ*B activation and reduced the VE-cadherin/*β*-catenin pathway. Taken together, these results suggest that hnRNPA2/B1 can regulate LPS-induced EC damage through regulating the NF-*κ*B and VE-cadherin/*β*-catenin pathways.

## 1. Introduction

Sepsis is a life-threatening organ dysfunction caused by a dysregulated host response to infection [1]. Septic shock, the most severe clinical manifestation of sepsis, is often accompanied by severe hypotension, microvascular perfusion insufficiency, and tissue damage, leading to multiple organ dysfunctions [2–4].

Microcirculation destruction, in which endothelial activation plays an important role, is a common pathogenic mechanism of sepsis [5, 6]. The vascular barrier is mainly composed of endothelial cells (ECs), cell-cell intercellular junctions, and extracellular components. Under normal conditions, the endothelial barrier is in a semipermeable state, and substances in the blood can be transferred to peripheral tissues. EC integrity is necessary to maintain vascular homeostasis and tissue fluid balance. EC injury triggered by the inflammatory response may lead to increased vascular permeability, protein leakage, inflammatory cell infiltration, uncontrollable tissue edema, and microthrombosis [7–9].

Lipopolysaccharide (LPS), the major component of the outer membrane of gram-negative bacteria, elicits inflammatory responses in immune and nonimmune cells, including ECs [10, 11]. Vascular ECs stimulated with LPS are activated by TLR4, CD14, and the MD-2 receptor complex, releasing a large number of proinflammatory factors and upregulating endothelial adhesion molecules, resulting in the loss of endothelial integrity, thus, leading to increased endothelial hyperpermeability, barrier function destruction, and even organ dysfunction [12–14].

Heterogeneous nuclear ribonucleoprotein A2/B1 (hnRNPA2/B1), a member of the hnRNP family of proteins, has been implicated in mRNA splicing, nuclear-cytoplasmic transport, posttranscriptional regulation, and the regulation of chromosomal stability [15–18]. hnRNPA2/B1 contributes to cell biological functions by regulating cell metabolism, migration, invasion, and proliferation and mitochondrial stress [19–21]. hnRNPA2/B1 was reported to play a key role in the cell cycle by regulating transcription levels of the CDK inhibitors p16INK4a, P21, and P27 [22, 23]. A recent study showed that hnRNPA2/B1 knockout reduced the expression of E-cadherin and promoted the EMT progression in breast cancer [24]. In addition, hnRNPA2/B1 protein was involved in peripheral mechanisms of tolerance restoration and attenuated inflammation in autoimmune endocrine diseases [25].

Nevertheless, the role of hnRNPA2/B1 in endothelial barrier function during the inflammatory process is unknown. It is also unclear whether hnRNPA2/B1 affects the LPS-induced vascular inflammatory response in sepsis. In the present study, we aimed at investigating the potential function and molecular mechanism of hnRNPA2/B1 in endothelial barrier function using an in vitro cell culture model in HUVECs. We report the effect of hnRNPA2/B1 on HUVEC permeability and the inflammatory response. Our results demonstrated the distinct role of hnRNPA2/B1 and its molecular mechanism in endothelial injury during sepsis.

## 2. Materials and Methods

### 2.1. Cell Culture and Reagents

HUVECs were cultured in high-glucose DMEM (HyClone, Logan, USA) supplemented with 10% (*v*/*v*) FBS (Gibco, Carlsbad, USA) and 1% (*v*/*v*) streptomycin/penicillin (Gibco) and maintained under 5% CO_2_ at 37°C. The cells were passaged with trypsin-EDTA, trypsin-neutralizing solution, and HEPES buffer every 3-4 days.

HUVECs were pretreated with 50 nM siRNA or 2.5 *μ*g plasmid for 48 h, followed by exposure to 1 *μ*g/ml LPS for 0 h, 6 h, and 12 h.

### 2.2. hnRNPA2/B1 siRNA and Plasmid Transfection

HUVECs at a density of 5 × 10^5^/well were seeded in 6-well culture plates and cultured for 24 h. The cells were transfected with 50 nM siRNA (hnRNPA2/B1 siRNA or NC siRNA; GenePharma Research, Shanghai, USA) or 2.5 *μ*g plasmid (pcDNA3.1(-)-Myc-6His or pcDNA3.1(-)-hnRNPA2/B1-Myc-6His) and 7.5 *μ*L of LipofectamineTM 3000 transfection reagent (Invitrogen, Carlsbad, CA, USA) per well for 24 h. The hnRNPA2/B1 siRNA sequences are shown in Table S1.

### 2.3. RT-qPCR

Total RNA was extracted from cells using TRIzol reagent (Invitrogen) according to the manufacturer's instructions. RT-qPCR was conducted with the Roche LightCycler 480II real-time PCR system (Roche) to detect the level of RNA transcripts. The primers used for RT-qPCR are shown in Table S2. All the data were analyzed using *β*-actin as an internal control. Relative copy numbers of target genes were determined using the 2^-*ΔΔ*Ct^ method.

### 2.4. Western Blotting

HUVECs were collected and lysed using a cell lysis solution containing complete protease inhibitors. Protein concentrations were determined using a BCA protein assay kit (Pierce, USA). Briefly, proteins were separated by 10% SDS-PAGE and transferred to polyvinylidene fluoride membranes (Hybond-P; GE Healthcare, Singapore). The membranes were incubated for 1 h in 5% nonfat milk and Tris-buffered saline with Tween-20, which was followed by an overnight incubation at 4°C with primary antibodies against the following: GAPDH (5174, Cell Signaling Technology), hnRNPA2/B1 (ab31645, Abcam), VE-cadherin (ab33168, Abcam), *β*-catenin (ab325728, Abcam), ICAM-1 (BBA3, R&D Systems), NF-*κ*B p65 (ab16502, Abcam), and p-NF-*κ*B p65 (ab86299, Abcam). The membranes were then incubated with goat anti-mouse IgG H&L (HRP) (ab6789, Abcam) or goat anti-rabbit IgG H&L (HRP) (ab6721, Abcam) for 2 h at room temperature. Proteins were visualized with an enhanced chemiluminescence kit (Pierce, USA) on a ChemiDocTM XRS+ system (Bio-Rad). The densities of the protein bands were quantified by ImageJ software using GAPDH as a reference.

### 2.5. Endothelial Permeability Measurement

#### 2.5.1. TEER Measurement

HUVECs at a density of 1 × 10^5^ cells/cm^2^ were seeded onto 0.4-*μ*m polycarbonate membrane filters inside 24-well Transwell cell culture chambers (Corning Costar, Cambridge, USA) and then treated with LPS and siRNA as described. TEER was measured using a MERSST×01 electrode according to the manufacturer's instructions (EMD Millipore Corporation, Billerica MA). TEER values were calculated by subtracting the blank value with the filter and multiplying by the surface area of the filter [26].

#### 2.5.2. FITC-Dextran Permeability Assay

Endothelial permeability was studied by measuring the apical-to-basolateral flux of 4-kDa FITC-dextran (Sigma-Aldrich). Briefly, after the cells had been treated for 12 h with LPS, the upper chambers were filled with 2.5 mg/mL FITC-dextran and incubated at 37°C for 2 h. One hundred microliters of medium from the basolateral chamber was transferred to a 96-well plate, and fluorescence was detected with a microplate fluorospectrophotometer at 520 nm.

### 2.6. ELISA

The levels of IL-1*β*, IL-6, and TNF-*α* in HUVEC culture supernatant were measured according to the manufacturer's protocol (MultiSciences Biotech Co., Ltd., China). The absorbance value at a wavelength of 450 nm was measured with a TriStar^2^ LB 942 Multimode Microplate Reader (Berthold Technologies, Germany).

### 2.7. Immunofluorescence

HUVECs were fixed with 4% formaldehyde and incubated with PBS containing 0.1% Triton X-100 (PBS-BT) and 5% normal serum for 1 h at room temperature. The solution was then removed and replaced with a solution of *β*-catenin (ab325728, Abcam) diluted 1 : 200. After incubation for 2 h at room temperature, the samples were rinsed and further incubated for 1 h with donkey anti-rabbit IgG H&L (Alexa Fluor® 488) (ab150073, Abcam) diluted 1 : 250. Cells were rinsed in PBST, followed by the addition of 1 *μ*g/mL DAPI (62248, Thermo Scientific). Samples were visualized using a Nikon Ts2-FL microscope (Nikon, Japan).

### 2.8. Statistics Analysis

All data are expressed as the mean ± standard deviations and were analyzed by one-way analysis of variance (ANOVA) with Bonferroni's multiple comparison test. At least three independent experiments were performed per treatment. Differences with a *p* value < 0.05 were considered statistically significant. Graphs were drawn using GraphPad Prism (version 8.0 for Windows, GraphPad Software Inc., San Diego, CA, USA).

## 3. Results

### 3.1. hnRNPA2/B1 Was Elevated in LPS-Stimulated HUVEC Cells

**Figure 1:**
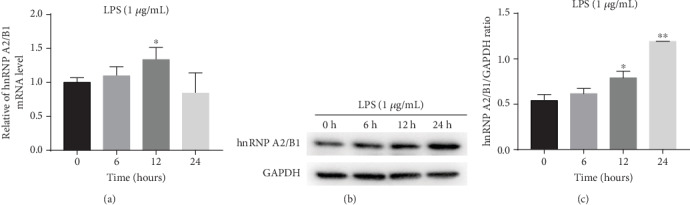
hnRNPA2/B1 expression in LPS-stimulated HUVECs. HUVECs were treated with 1 *μ*g/mL LPS for 0 h, 6 h, 12 h, and 24 h. (a) hnRNPA2/B1 mRNA expression levels were quantified by qRT-PCR, and *β*-actin was used as a normalization control. (b, c) hnRNPA2/B1 protein levels were measured by Western blotting, and GAPDH was used as an internal control. ^∗^*p* < 0.05, ^∗∗^*p* < 0.01, ^∗∗∗^*p* < 0.001.

To explore the effect of hnRNPA2/B1 on the LPS-stimulated HUVECs, we first detected hnRNPA2/B1 expression. The HUVECs were treated with 1 *μ*g/mL of LPS for 0, 6, 12, and 24 h. hnRNPA2/B1 mRNA was quantified by RT-qPCR, and its protein level was analyzed by Western blotting. As shown in Figure 1, LPS treatment promoted the expression of hnRNPA2/B1 in a time-dependent manner. 6, 12, and 24 hours of LPS stimulation increased the level of hnRNPA2/B1 significantly. Compared with 0 h, the mRNA (Figure 1(a)) and protein expression (Figures 1(b) and 1(c)) of hnRNPA2/B1 significantly increased at 12 h, respectively.

### 3.2. Effect of hnRNPA2/B1 on LPS-Induced Endothelial Permeability Dysfunction

**Figure 2:**
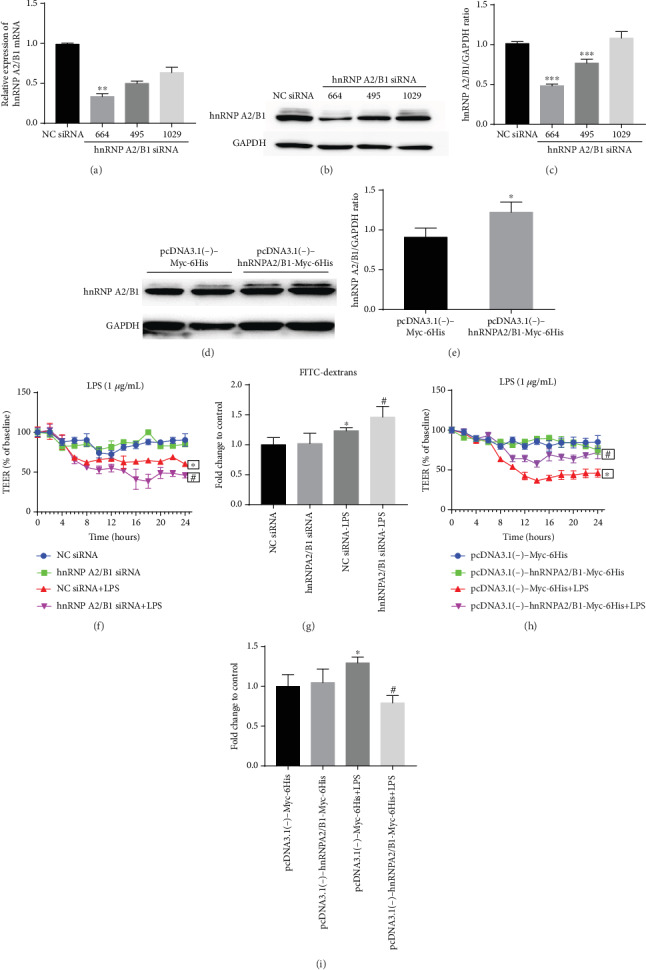
Effect of hnRNPA2/B1 on endothelial permeability in HUVECs. HUVECs were transfected with hnRNPA2/B1 siRNA/negative control (NC) siRNA or pcDNA3.1(-)-Myc-6His/pcDNA3.1(-)-hnRNPA2/B1-Myc-6His plasmid for 36 h and then stimulated with 1 *μ*g/mL LPS for different lengths of time. The hnRNPA2/B1 knockdown efficiency was determined by qRT-PCR (a) and Western blotting (b, c). The hnRNPA2/B1 overexpression efficiency was determined by Western blotting (d, e). (f, h) TEER values were measured every 2 h for 24 h of LPS stimulation. (g, i) Endothelial permeability was determined by FITC-dextran flux analysis 12 h after LPS administration. Data are expressed as mean ± SD (*n* = 3), ^∗^ vs. Negative control (siRNA/plasmid) *p* < 0.05, ^#^ vs. Negative control (siRNA/plasmid) + LPS*p* < 0.05.

To investigate the effect of hnRNPA2/B1 on LPS-induced endothelial injury in HUVECs, the cells were transfected with small interfering RNA (siRNA) against hnRNPA2/B1 (664, 495, and 1029) to knockdown hnRNPA2/B1 expression or negative control (NC) siRNA. hnRNPA2/B1 mRNA and protein levels were significantly decreased after gene knockdown (Figures 2(a)–2(c)). hnRNPA2/B1 siRNA-664 showed the highest knockdown efficiency and was used in subsequent experiments. Also, the HUVECs were transfected with pcDNA3.1(-)-hnRNPA2/B1-Myc-6His plasmid to upregulate the hnRNPA2B1 expression. hnRNPA2/B1 protein levels were significantly increased after gene overexpression (Figures 2(d) and 2(e)).

To verify the regulatory role of hnRNPA2/B1 in LPS-induced endothelial permeability, hnRNPA2/B1 siRNA-transfected and plasmid-transfected HUVECs were stimulated with 1 *μ*g/mL LPS for the indicated durations, and barrier function was then assessed by measuring the transendothelial electrical resistance (TEER). The TEER value six hours was lower after treatment with LPS than in the NC, suggesting that LPS stimulation significantly increased the permeability of HUVECs. Compared with NC siRNA-transfected HUVECs, hnRNPA2/B1 siRNA-transfected HUVECs exhibited a reduced TEER value after LPS stimulation, while TEER value were significantly higher in hnRNPA2B1-overexpression group than the control plasmid group, indicating that hnRNPA2/B1 suppression destroyed endothelial permeability and aggravated endothelial barrier dysfunction. However, the upregulation of hnRNPA2/B1 may attenuate the endothelial barrier damage (Figures 2(f) and 2(h)). Furthermore, endothelial monolayer permeability was determined by FITC-dextran Transwell assay. FITC-dextran permeation through the cell monolayer is used as a marker to determine endothelial permeability in vitro. FD4 fluorescence was detected in the lower chamber 12 h after LPS stimulation. Compared with that in NC samples, LPS treatment induced a significant increase in the leakage of 4-kDa FITC-dextran from the endothelial monolayer, and pretreatment with hnRNPA2/B1 appeared to gradually increase leakage after LPS stimulation for 12 h compared with that in NC siRNA-transfected cells treated with LPS. Compared with the negative control plasmid group, overexpression of hnRNPA2/B1 could reduce FITC-dextran leakage (Figures 2(g) and 2(i)). These results showed that the protective role of hnRNPA2/B1 on endothelial permeability in HUVEC monolayers in vitro.

### 3.3. Effect of hnRNPA2/B1 on LPS-Induced Inflammatory Response

**Figure 3:**
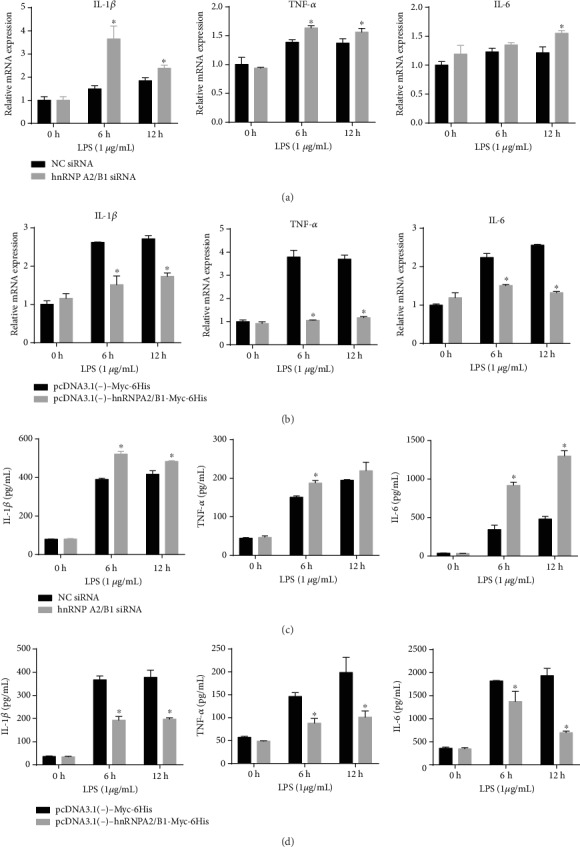
Effect of hnRNPA2/B1 on inflammatory cytokine expression in HUVECs. Twelve hours after LPS treatment, HUVECs and culture supernatant were collected. (a, c) The expression levels of IL-1*β*, TNF-*α*, and IL-6 were measured by RT-qPCR. *β*-Actin was used as a normalization control. (b, d) IL-1*β*, TNF-*α*, and IL-6 concentrations were assayed by ELISA. Data are expressed as mean ± SD (*n* = 3), ^∗^ vs. Negative control (siRNA/plasmid) *p* < 0.05.

To explore the role of hnRNPA2/B1 in LPS-induced endothelial inflammation, we detected the expression of inflammatory factors. As shown in Figure 3, LPS treatment increased IL-6, IL-1*β*, and TNF-*α* expression compared with that in NC siRNA-transfected HUVECs, and hnRNPA2/B1 depletion remarkably increased the levels of IL-6, IL-1*β*, and TNF-*α*. On the other hand, hnRNPA2/B1 overexpression inhibited the LPS-induced IL-6, IL-1*β*, and TNF-1*α* expression. This evidence confirmed that hnRNPA2/B1 inhibition boosted proinflammatory factor expression in the LPS-induced endothelial inflammatory response. HnRNPA2/B1 may protect the endothelium against inflammatory response.

### 3.4. Effects of hnRNPA2/B1 on LPS-Induced Endothelial Injury in HUVECs Are VE-Cadherin/*β*-Catenin Dependent

**Figure 4:**
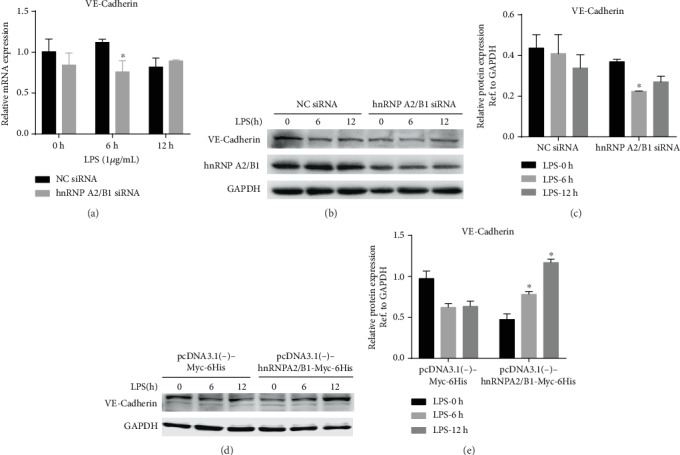
Effect of hnRNPA2/B1 on the expression of adherens junction molecules in LPS-stimulated HUVECs. HUVECs were transfected with hnRNPA2/B1 siRNA/negative control (NC) siRNA or pcDNA3.1(-)-Myc-6His/pcDNA3.1(-)-hnRNPA2/B1-Myc-6His plasmid for 36 h and then stimulated with 1 *μ*g/mL LPS for 0, 6, and 12 h. The mRNA and protein were extracted, and VE-cadherin and GAPDH mRNA and protein levels were detected by RT-qPCR and Western blotting, respectively. Data are expressed as mean ± SD (*n* = 3), ^∗^ vs. Negative control (siRNA/plasmid) *p* < 0.05.

The expression of adhesion junction molecules is involved in maintaining endothelial integrity and permeability. To explore the action underlying the effects of hnRNPA2/B1 on endothelial permeability, we further evaluated the expression of adhesion junction proteins in LPS-treated HUVECs. LPS treatment for 6 h significantly decreased the expression of VE-cadherin. hnRNPA2/B1 siRNA treatment decreased VE-cadherin expression compared with that in NC siRNA-transfected HUVECs treated with LPS (Figures 4(a)–4(c)). Interestingly, overexpression of hnRNPA2/B1 promoted VE-cadherin expression compared with that in negative control plasmid-transfected HUVECs (Figures 4(d) and 4(e)). These results indicate that hnRNPA2/B1 maintains endothelial permeability associated with VE-cadherin.

**Figure 5:**
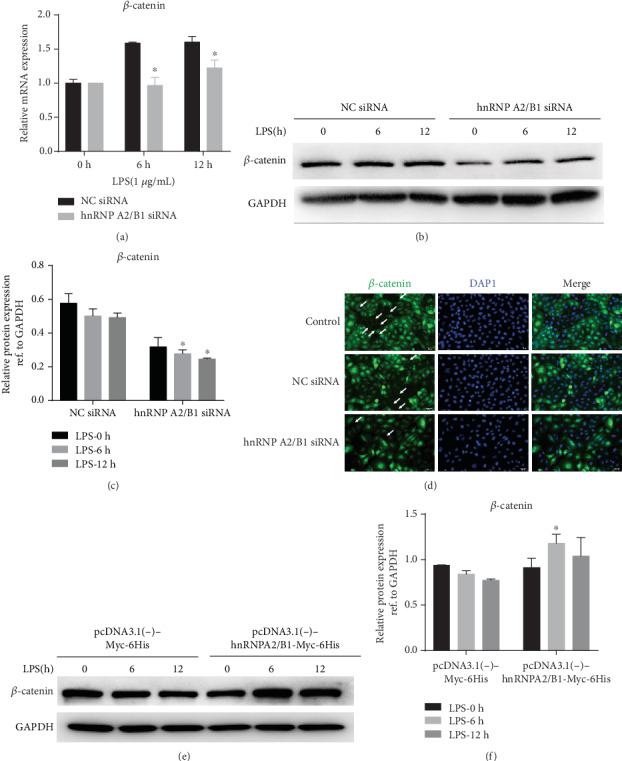
The effect of hnRNPA2/B1 reverses the LPS-induced downregulation of *β*-catenin and endothelial hyperpermeability in HUVECs. Twelve hours after LPS treatment, HUVECs, and culture supernatant were collected. (a) The mRNA expression level of *β*-catenin was measured by RT-qPCR. *β*-Actin was used as a normalization control. (b, c, e, f) The protein expression level of *β*-catenin was detected by Western blotting. (d) Immunofluorescent staining for *β*-catenin (green) in HUVECs stained with DAPI (blue). Arrows indicate areas of cell-cell adhesion. Scale bars, 50 *μ*m. Data are expressed as mean ± SD (*n* = 3), ^∗^ vs. Negative control (siRNA/plasmid) *p* < 0.05.

The cytoplasmic part of VE-cadherin is linked to armadillo repeat genes, including *β*-, *γ*-, *α*-, and p120-catenins. *β*-Catenin is thought to play a dual role in modulating endothelial barrier function. At adherens junctions (AJs), *β*-catenin stabilizes VE-cadherin-mediated cell-cell adhesion by anchoring the cytoplasmic tail of VE-cadherin to the actin cytoskeleton [27]. We demonstrate that *β*-catenin plays an important role in maintaining endothelial homeostasis. Treatment with hnRNPA2/B1 siRNA significantly aggravated the LPS-mediated loss of *β*-catenin, a binding partner of VE-cadherin, at the mRNA and protein levels (Figures 5(a)–5(c)). Immunostaining for *β*-catenin showed a linear staining pattern at the cell-cell boundary of HUVECs under control conditions in confluent endothelial monolayers. Incubation with LPS for 12 h led to intercellular gap formation and a pronounced loss of *β*-catenin staining at cell borders. Pretreatment with hnRNPA2/B1 siRNA significantly increased the loss of *β*-catenin (Figure 5(d)). However, hnRNPA2/B1 overexpression obviously enhances the expression of *β*-catenin (Figures 5(e) and 5(f)). Taken together, these findings suggest that hnRNPA2/B1 reverses LPS-induced downregulation of VE-cadherin/*β*-catenin and endothelial hypermeability. Thus, we concluded that the effect of hnRNPA2/B1 on LPS-induced endothelial barrier protection in HUVECs is VE-cadherin/*β*-catenin dependent.

### 3.5. Knockdown of hnRNPA2/B1 Promoted LPS-Induced NF-*κ*B Activation

**Figure 6:**
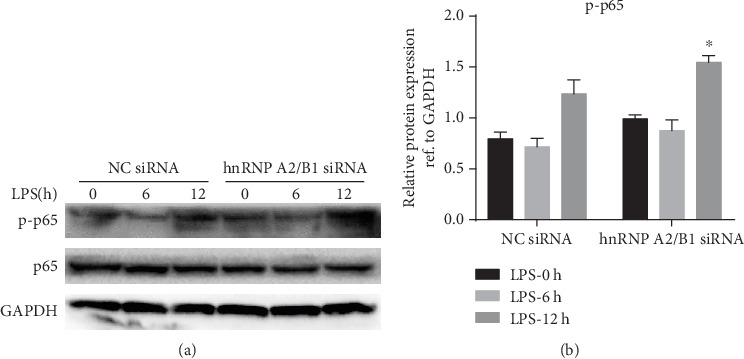
Effect of hnRNPA2/B1 knockdown on LPS-induced NF-*κ*B signaling activation. HUVECs transfected with siRNA (hnRNPA2/B1 or negative control) were cultured for 36 h and then treated with 1 *μ*g/mL LPS for 0, 6, and 12 h. (a, b) NF-*κ*B phosphorylation was examined by Western blotting, and GAPDH was used as an internal reference. Data are expressed as mean ± SD (*n* = 3), ^∗^ vs. NC siRNA *p* < 0.05.

To determine whether hnRNPA2/B1 regulates the inflammatory response signaling pathway, we detected NF-*κ*B signaling activation. Western blotting was performed to detect the levels of p65 phosphorylation. The results showed that hnRNPA2/B1 depletion remarkably increased the levels of p-p65 (Figures 6(a) and 6(b)) compare to those under NC conditions. Consequently, we concluded that hnRNPA2/B1 depletion activates NF-*κ*B signaling to promote proinflammatory cytokine expression during LPS-treated endothelial inflammation.

## 4. Discussion

Our findings suggest that hnRNPA2/B1 is involved in LPS-induced hyperpermeability and the inflammatory response. Moreover, hnRNPA2/B1 suppression increased the LPS-induced upregulation of TNF-*α*, IL-1*β*, and IL-6 in the cell culture supernatant. Furthermore, hnRNPA2/B1 may prevent the downregulation of VE-cadherin/*β*-catenin signaling in LPS-stimulated HUVECs. In addition, hnRNPA2/B1 inhibition induced NF-*κ*B activation. Therefore, we speculate that hnRNPA2/B1 exerts its protective effect on LPS-induced hyperpermeability and inflammatory damage in HUVECs by inhibiting NF-*κ*B activation and subsequently preventing decreased VE-cadherin/*β*-catenin signaling.

Sepsis is a major cause of death in developed and developing countries [28]. Endothelial barrier function, which is particularly critical for the pathogenesis of sepsis, is highly integrated within the systemic inflammatory response. Endothelial functions, including vasoregulation, barrier function, inflammation, and hemostasis, are regulated by sepsis. Endothelial barrier dysfunction is also a key to the pathogenesis of sepsis-related complications, such as acute lung injury [7, 26–28]. Vascular leakage and edema appear in septic patients with endothelial hyperpermeability and lead to a decrease in effective blood volume and blood pressure, greatly increasing the risk of death [6, 29]. Therefore, it is crucial to protect against vascular endothelial integrity in sepsis treatment. In our study, we silenced the hnRNPA2/B1 gene and observed that knockdown of hnRNPA2/B1 significantly reduced the TEER value and increased FITC-dextran permeability during LPS-induced endothelial damage, leading to endothelial barrier dysfunction. However, the upregulation of hnRNPA2/B1 may reverse LPS-induced endothelial barrier damage effect. These results suggest that hnRNPA2/B1 has a protective effect on endothelial permeability changes in HUVECs induced by LPS.

The vascular barrier consists of ECs, cell-cell junctions, and extracellular components [7]. ECs are connected by two types of adhesion complexes that maintain cell-cell interactions: AJs and tight junctions (TJs). AJs are involved in multiple functions, including the establishment and maintenance of cell-cell adhesion, actin cytoskeleton remodeling, intracellular signaling, and transcriptional regulation [30–33]. The vascular endothelial adhesion protein VE-Cadherin plays a key role in maintaining and regulating the stability of endothelial AJs [34]. Studies have shown that VE-Cadherin antibodies can disrupt junctions between ECs, leading to increased permeability and the extravasation of leukocytes [35]. In addition, VE-Cadherin overexpression could enhance adhesion function, stabilizing the endothelial barrier and reducing vascular permeability and leukocyte extravasation [36]. Under normal conditions, the endothelial barrier is semipermeable and allows fluids and solutes to be transported from the blood to tissues. However, the destruction of the endothelial barrier results in the enhanced levels of proteins in the circulation and edema [7]. We evaluated the effect of hnRNPA2/B1 on the expression of adhesion junction proteins in LPS-treated HUVECs. hnRNPA2/B1 siRNA treatment decreased VE-cadherin expression compared with that in NC siRNA-transfected HUVECs treated with LPS. Overexpression of hnRNPA2/B1 promoted VE-cadherin expression compared with that in negative control plasmid-transfected HUVECs. These results indicate that hnRNPA2/B1 maintains endothelial permeability associated with VE-cadherin.

In addition, VE-cadherin is involved in cell proliferation, apoptosis, and transcriptional regulation [27]. VE-cadherin can indirectly participate in gene transcription by inhibiting the activity of FoxO1, leading to the upregulation of claudin-5 [37]. Studies have shown that VE-cadherin directly binds p120-catenin, *β*-catenin, and *γ*-catenin through its cytoplasmic domain, which is essential for maintaining the integrity of adhesive connections. In the AJ complex, VE-cadherin binds p120-catenin and *β*-catenin, which are known mediators of gene transcription [38]. Treatment of ECs with the permeability-inducing factor thrombin induced the translocation of p120-catenin and *β*-catenin into the nucleus and the expression of *β*-catenin target genes, which indicated that the binding of p120-catenin and *β*-catenin to the AJ complex may reduce the signaling pathways of these molecules. Multiple studies suggest the role of *β*-catenin-mediated Wnt transcription in EC biology and vascular development [39, 40]. We demonstrate that *β*-catenin plays an important role in maintaining endothelial homeostasis. Treatment with hnRNPA2/B1 siRNA significantly aggravated the LPS-mediated loss of the VE-cadherin-binding partner *β*-catenin at the mRNA and protein levels. Immunostaining for *β*-catenin showed a linear staining pattern at the cell-cell boundary of HUVECs under control conditions in confluent endothelial monolayers. Incubation with LPS for 12 h led to the intercellular gap formation and a pronounced loss of *β*-catenin staining at cell borders. Pretreatment with hnRNPA2/B1 siRNA significantly increased the loss of *β*-catenin. Thus, we conclude that hnRNPA2/B1 suppression contributes to the LPS-induced downregulation of VE-cadherin/*β*-catenin and endothelial hyperpermeability. In addition, hnRNPA2/B1 overexpression obviously enhances the expression of *β*-catenin. Therefore, we speculate hnRNPA2/B1 protecting HUVECs against LPS-induced endothelial barrier is VE-cadherin/*β*-catenin dependent.

Endothelial dysfunction may be a deleterious consequence of excessive cytokine production. Mediators such as TNF-*α* activate signaling events that culminate in cytoskeletal contraction and increase microvascular permeability [41]. In addition, activated neutrophils release neutrophil extracellular traps and bactericidal proteins, which have been shown to enhance cytotoxic effects on ECs [42, 43]. hnRNPA2/B1 depletion remarkably increased the levels of IL-6, IL-1*β*, and TNF-*α* under LPS stimulation. This evidence confirmed that hnRNPA2/B1 inhibition triggers proinflammatory cytokine expression in the LPS-induced endothelial inflammatory response. Endothelial NF-*κ*B activation plays a key role in the cascade of events leading to EC dysfunction in sepsis [44, 45]. Blockade of NF-*κ*B activation resulted in reduced iNOS expression and nitrosative stress and attenuated eNOS downregulation, all of which have a beneficial protective effect in ECs [46]. Blockade of cytokine signaling by inhibiting NF-*κ*B activation resulted in decreased inflammation and endothelial permeability as well as improved endothelial barrier function [47]. In our present study, we observed that hnRNPA2/B1 depletion remarkably increased the levels of p65 phosphorylation. Consequently, we concluded that hnRNPA2/B1 depletion activates NF-*κ*B signaling to promote proinflammatory cytokine expression during LPS-induced endothelial inflammation.

## 5. Conclusions

Collectively, our results demonstrate for the first time that hnRNPA2/B1 suppression caused significant damage to endothelial barrier integrity in parallel with the downregulation of VE-cadherin/*β*-catenin and NF-*κ*B activation. Furthermore, hnRNPA2/B1 upregulation protected the HUVECs against LPS-induced ECs damage. These results strongly suggest that hnRNPA2/B1 has a protective effect and plays an important role in maintaining the integrity of AJs and suppressing the inflammatory response in ECs. Therefore, hnRNPA2/B1 is a remarkable molecular therapeutic target for the regulation of vascular hyperpermeability during sepsis, and targeting hnRNPA2/B1 may benefit critically ill patients.

## Data Availability

The data used to support the findings of this study are available from the corresponding author upon request.

## Supplementary Materials

Supplementary 1 Table S1: siRNA sequence for hnRNPA2/B1 and negative control. Table S2: primer list for RT-qPCR.

Supplementary 2 Figure S1: the mRNA level of hnRNPA2/B1.
