# Supplementary figures and images for: hnRNPA2/B1 Ameliorates LPS-Induced Endothelial Injury through NF-κB Pathway and VE-Cadherin/β-Catenin Signaling Modulation In Vitro
Source: Mediators Inflamm. 2020 May 30;2020:6458791. doi: 10.1155/2020/6458791 (PMC7277030; doi:10.1155/2020/6458791)

## Slide 1
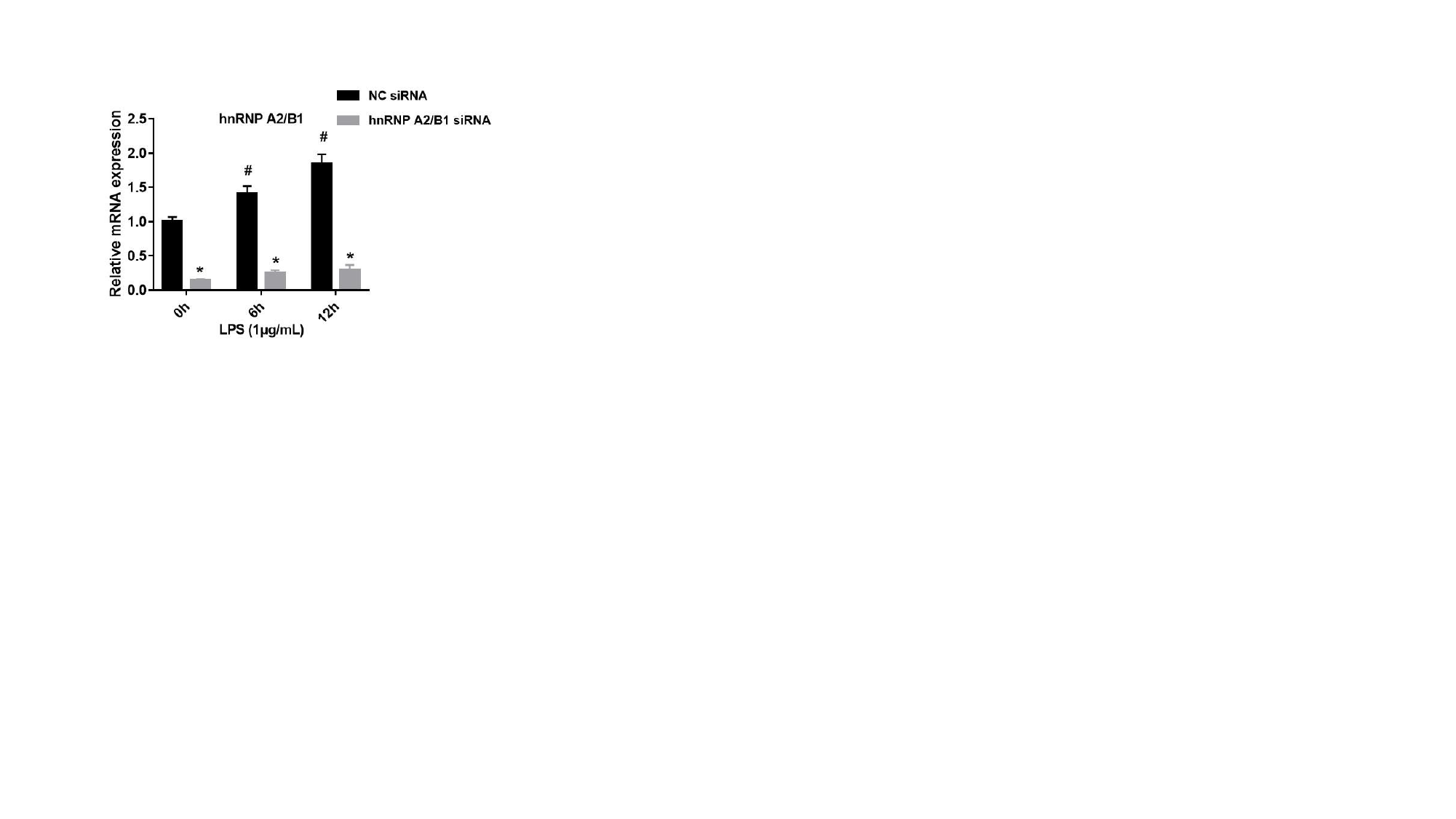

Supplement: Supplementary 1 — Table S1: siRNA sequence for hnRNPA2/B1 and negative control. Table S2: primer list for RT-qPCR. [file 6458791.f1.pptx]
