# Supplementary material for: hnRNPA2/B1 Ameliorates LPS-Induced Endothelial Injury through NF-κB Pathway and VE-Cadherin/β-Catenin Signaling Modulation In Vitro
Source: Mediators Inflamm. 2020 May 30;2020:6458791. doi: 10.1155/2020/6458791 (PMC7277030; doi:10.1155/2020/6458791)
Supplement: Supplementary 2 — Figure S1: the mRNA level of hnRNPA2/B1. [file 6458791.f2.docx]

**Table S1.** siRNA sequence for hnRNP A2/B1 and negative control

| **Gene** | | **Sense (5’-3’)** | **Antisense (5’-3’)** |
| --- | --- | --- | --- |
| hnRNP A2/B1-homo-495 | GGGCUCAUGUAACUGUGAATT | | UUCACAGUUACAUGAGCCCTT |
| hnRNP A2/B1-homo-664 | | CCAGUAUCCUGUGGAUAAATT | UUUAUCCACAGGAUCAUGGTT |
| hnRNP A2/B1-homo-1029 | | GAGGUGGUUAUGACAACUATT | UAGUUGUCAUAACCACCUCTT |
| Negative Control | UUCUCCGAACGUGUCACGUTT | | ACGUGACACGUUCGGAGAATT |

**Table S2.** Primers list for RT‐qPCR.

| **Gene** | **Forward primer (5’-3’)** | **Reverse primer(5’-3’)** |
| --- | --- | --- |
| hnRNPA2/B1 | CTGTAGCAAGAGAGGAGTCTGGA | CATACCCATTATAGCCATCCCCA |
| IL-1β | ACTACAGCAAGGGCTTCAGG | CTTTTTTGCTGTGAGTCCCG |
| IL-6 | CAGAAAACAACCTGAACCTT | GCTTGTTCCTCACTACTCTC |
| TNF-α | CAGACTTCCTTGAGACACGG | CAAGGCAGCTCCTACATTGG |
| ICAM-1 | GCAATGTGCAAGAAGATAGCCA | GTGAGGAAGGTTTTAGCTGTTGACTG |
| VE-Cadherin | GGCAAGATCAAGTCAAGCGTG | ACGTCTCCTGTCTCTGCATCG |
| β-catenin | GCTGATTTGATGGAGTTGGAC | AGGAGCTGTGGTAGTGGCACCAGAATGGAT |
| β-actin | TTCTACAATGAGCTGCGTGTG | GGGGTGTTGAAGGTCTCAAA |
